# Tachyarrhythmias in Congenital Heart Diseases: From Ion Channels to Catheter Ablation

**DOI:** 10.3390/jcdd9020039

**Published:** 2022-01-24

**Authors:** Victor Waldmann, Jean-Baptiste Guichard, Eloi Marijon, Paul Khairy

**Affiliations:** 1 Adult Congenital Heart Disease Medico-Surgical Unit, European Georges Pompidou Hospital, 75015 Paris, France; 2 Pediatric and Congenital Cardiology Medico-Surgical Unit, Necker Enfants Malades Hospital, 75015 Paris, France; 3 Faculté de Médicine, Université de Paris, 75006 Paris, France; eloi.marijon@aphp.fr; 4 Cardiology Department, University Hospital of Saint-Étienne, 42000 Saint-Étienne, France; j.baptiste.guichard@chu-st-etienne.fr; 5 Department of Medicine, Montreal Heart Institute Research Center, Université de Montréal, Montreal, QC H1T 1C8, Canada; 6 Department of Cardiology, European Georges Pompidou Hospital, 75015 Paris, France; 7 Electrophysiology Service and Adult Congenital Heart Centre, Montreal Heart Institute, University of Montreal, Montreal, QC H1T 1C8, Canada; paul.khairy@umontreal.ca

**Keywords:** congenital heart disease, arrhythmia, catheter ablation, pathophysiology, substrate

## Abstract

Major advances in pediatric cardiology in recent decades, especially surgical techniques, have resulted in an increasing number of patients with congenital heart disease (CHD) surviving to adulthood. This has generated new challenges, particularly with regards to the late onset of complex arrhythmias. Abnormal anatomy, surgical scarring, chronic hypoxemia, hemodynamic compromise, neuro-hormonal abnormalities, and genetic factors can all contribute to creating a unique substrate for arrhythmia development. This review attempts to synthesize the current state of knowledge spanning the spectrum from underlying mechanisms of arrhythmias in patients with congenital heart disease to current ablative strategies. We discuss existing knowledge gaps and highlight important areas for future research.

## 1. Introduction

As a result of major advances achieved in the past decades in pediatric cardiology and surgical techniques regarding anatomical correction of congenital defects, most patients with congenital heart disease (CHD) now reach adulthood, such that the population of survivors is increasing and aging [1]. This success is, however, tempered by the onset of late complications, including arrhythmias that are a major source of morbidity. Projections indicate that 50% of 20-year-old subjects will experience an atrial tachyarrhythmia during their lifespan [2]. Abnormal anatomy, post-surgical scarring, and systemic factors contribute to establishing a unique substrate for arrhythmia development. In this review, we summarize the specific substrates, triggers, and modulators for arrhythmias in CHD (Table 1), discuss current management strategies, including catheter ablation, and offer future perspectives (Table 2).

## 2. Arrhythmias Pathophysiology and Genesis in Congenital Heart Disease

### 2.1. Substrates for Arrhythmias in Congenital Heart Disease

#### 2.1.1. Congenital Heart Disease-Related Substrate

Although in most cases arrhythmias in CHD are an acquired condition resulting from surgical scars and other chronic contributing factors, cardiac arrhythmias may also be related to the structural malformation itself. The development of conduction pathways may be impacted by the embryological abnormalities responsible for CHD, and the atrioventricular (AV) node and the His bundle may be displaced beyond the confines of Koch’s triangle. Atypical AV nodal reentrant tachycardia (AVNRT) has for example been reported in patients with complex CHD, in particular cc-TGA and univentricular hearts, with displaced AV nodes and slow pathways [3,4]. The prevalence of AVNRT across the various forms of CHD remains, however, poorly characterized.

In some patients, the structural cardiac malformation can be accompanied by accessory or duplicate AV connections with the potential for reentrant tachyarrhythmias [5]. The most glaring example is the Ebstein anomaly, where accessory pathways have been reported in 10 to 38% of patients [6]. When present, multiple accessory pathways along the abnormal tricuspid annulus are found in up to 50% of patients, sometimes with complex insertion patterns. Accessory pathways are also prevalent in patients with Ebsteinoid malformations of the tricuspid valve in the setting of congenitally corrected transposition of the great arteries (cc-TGA). Additional types of CHD associated with an increased prevalence of accessory pathway include heterotaxy syndromes, AV septal defects, and some forms of univentricular hearts.

Duplicate AV connections were first described by Mönckeberg in 1913, with two separate coexisting AV nodes, usually called “twin AV nodes”. In the presence of a sling of tissue connecting the two AV conduction systems, so-called Mönckeberg sling [7], a macroreentrant circuit can arise with a reciprocating tachycardia that courses antegrade by one AV nodal pathway and retrograde via the second AV nodal pathway [8]. This scenario occurs most commonly in patients with a constellation of congenital heart defects, i.e., AV discordance, malaligned complete AV septal defect, and right or left atrial isomerism.

#### 2.1.2. Post-Operative Substrate

Reentrant tachycardias are frequently encountered after surgical repair of a wide variety of types of CHD. The initiation and maintenance of a reentrant arrhythmia require the presence of myocardial tissue with adjacent tissue having altered electrophysiological properties. Suture lines, patches or prosthetic material provide a core of inexcitable tissue that creates a central area of block, with the potential for reentrant circuits to form around these obstacles. Moreover, delayed conduction across areas of abnormal myocardial tissue allows for the circulating wave fronts to reach adjacent substrates that are no longer refractory, thereby permitting tachycardia circuits to be sustained.

At the atrial level, circuits encountered vary according to the anatomic defect and type of surgical repair. On the whole, cavo-tricuspid isthmus-dependent circuits remain the most common in patients with CHD [9]. Incisional intra-atrial reentrant tachycardia (IART), in particular around a right lateral atriotomy, is the second most common circuit. In other forms of CHD, such as in patients with older-style Fontan surgery (i.e., right atrium to pulmonary artery connections), long-term hemodynamic stress results in markedly abnormal atrial myocardium prone to various IART circuits around scar areas scattered in the atria. A substantial proportion of arrhythmias encountered have a focal activation pattern and are thought to be micro-reentrant circuits by virtue of their mode of induction and termination and response to pacing maneuvers. The term non-automatic focal atrial tachycardia (NAFAT) is commonly used in this setting to distinguish these arrhythmias from the more standard focal arrhythmias that are due to abnormal automaticity.

Similarly, the propensity to develop ventricular arrhythmias is influenced by the presence of a ventriculotomy and/or a patch or conduit inserted in the ventricle. The most studied example is the tetralogy of Fallot, where four potential anatomical isthmuses that could sustain macroreentrant ventricular tachycardia circuits are well characterized: isthmus 1, bordered by the tricuspid annulus and a right ventricular incision; isthmus 2, between the right ventricular incision and pulmonary valve; isthmus 3, between the pulmonary valve and ventricular septal defect patch; and isthmus 4, between the ventricular septal defect patch and tricuspid annulus. Isthmus 3 is the narrowest and appears to be most commonly implicated substrate in ventricular tachycardia circuits [10]. Progress in surgical techniques from a classical transventricular to a transatrial-transpulmonary approach may eliminate or alter the geometry of isthmuses 1 and 2 but does not alter the presence of isthmuses 3 or 4.

Lastly, and more anecdotally, accessory AV pathways may be created by surgical intervention, as reported in some patients with tricuspid atresia after a Fontan–Björk procedure connecting the right atrial appendage to the right ventricular outflow tract [11].

#### 2.1.3. Cardiovascular Risk-Related Substrate

As the CHD population ages, it also appears that factors associated with atrial arrhythmias (mainly atrial fibrillation) in the general population, such as ageing, obesity, hypertension, obstructive sleep apnea, and male gender, are likewise associated with arrhythmias in patients with CHD. In a multicenter study of patients with heterogeneous forms of CHD and atrial arrhythmias, factors independently associated with atrial fibrillation were older age, number of cardiac surgeries, and traditional cardiovascular risk factors [12]. These factors contribute to electrical and structural atrial remodeling that promotes the genesis of atrial arrhythmias. The importance of considering associated conditions in patients with CHD is increasingly recognized, but while screening for and aggressively managing cardiovascular risk factors and coexisting comorbidities appears to impact favorably outcomes in non-CHD populations [13], comparable data specific to adults with CHD are lacking.

#### 2.1.4. Genetic Substrate

Appreciation of the role of genetics in the pathogenesis of CHD has increased at a rapid pace over the past 15 years. Epidemiological studies have suggested that a genetic cause can be identified in more than 20% of cases. Single-gene disorders are found in 3% to 5%, gross chromosomal anomalies/aneuploidy in 8% to 10%, and pathogenic copy number variants in 3% to 25% of those with CHD as part of a syndrome and in 3% to 10% among those with isolated CHD [14]. Genotype/phenotype correlations have revealed that genetic abnormalities could be associated with an increased risk of cardiac arrhythmias, and an interesting example is provided by mutations in the NKX2-5 gene that encodes a homeobox transcription factor known to be involved in a diverse set of congenital heart malformations. The most common clinical presentation associates atrial septal defect with AV block, with a high incidence of sudden cardiac death. Although the predominant cause of sudden death is thought to be related to conduction disorders, an increased risk of tachyarrhythmia is also reported [15]. Analyses of embryos showed a down-regulation and the abnormal expression of different gap junction proteins (GJas) as an underlying explanation for abnormalities in impulse propagation, and subsequently the development of arrhythmias. Reduced levels of GJa1 were shown to contribute to an increased risk of ventricular arrhythmias and sudden death, while reduced GJa5 levels were linked to atrial electrical instability with increased risk of atrial fibrillation [16]. Furthermore, a lateral distribution of gap junctions at myocyte junction borders is thought to affect dissipation of the cardiac impulse within the ventricular sink, prolonging its propagation and heightening the risk of arrhythmogenesis through micro-reentry circuits.

### 2.2. Triggers for Arrhythmias in Congenital Heart Diseases

#### 2.2.1. Abnormal Automaticity and Triggered Activity

**Figure 1:**
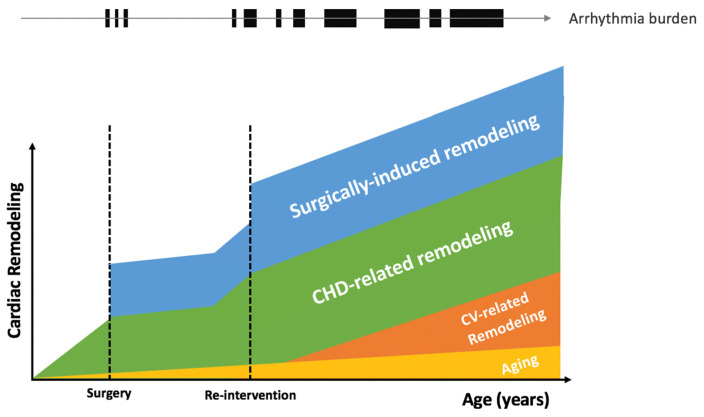
Cardiac remodeling and arrhythmia burden in congenital heart diseases. CHD, congenital heart disease; CV, cardiovascular.

Automaticity is the property of cardiac cells to generate spontaneous action potentials. It results from diastolic depolarization caused by a net inward current during phase 4 of the action potential. Under normal conditions, atrial and ventricular myocardial cells do not display spontaneous diastolic depolarization or automaticity. Afterdepolarizations are depolarizations that attend or follow the cardiac action potential and depend on preceding transmembrane activity for their manifestation. Early afterdepolarizations (EAD) interrupt or delay repolarization during phase 2 and/or phase 3 of the cardiac action potential, whereas delayed afterdepolarizations (DAD) occur after full repolarization [17]. While triggered activity and abnormal automaticity has not been specifically described in patients with CHD, the importance of progressive structural cardiac remodeling in CHD (Figure 1) is associated with electrical remodeling involving alteration of ion channels, pumps, and exchangers, that may be associated with increased abnormal automaticity and/or afterdepolarizations [18]. While focal arrhythmias in CHD are often related to presumed micro reentry mechanisms (NAFAT) [19], triggered events may also cause automatic tachycardias and arrhythmias with focal activation pattern account for 5–10% of all regular atrial arrhythmias [9,20]. Abnormal impulses also give rise to premature beats, which can precipitate tachyarrhythmias by initiating reentrant circuits.

#### 2.2.2. Hemodynamic Alterations

The hemodynamic abnormalities associated with CHD contribute importantly to ventricular and atrial structural remodeling. Left ventricular outflow tract obstruction is a classic example of the role of hemodynamic changes on structural remodeling, with resultant left ventricular overload and increased fibrotic process [21]. In patients with ventricular septal defects, the left ventricular hypertrophy and extent of fibrosis are independently associated with sudden death [22]. Years of palliation before surgical repair, non-pulsatile subpulmonary perfusion, single or systemic right ventricles, obstruction to conduits or leaks, and valvular regurgitation are other specific elements that may contribute to hemodynamic derangements in patients with CHD. From a chronological point of view, rather than ageing itself, the longer the time interval prior to surgical repair, the higher the long-term arrhythmia risk. Histology and immunohistochemistry analyses of myocardial tissue resected from 65 CHD patients who underwent cardiac surgery revealed that extent of fibrosis, myocyte diameter, capillary distance, and CD45-positive cell infiltration were correlated with overload duration, and that this time-course related remodeling was greater in patients with a history of arrhythmias [23].

Hemodynamic and arrhythmic complications are so intimately linked that it is strongly recommended for adults with CHD and new-onset or worsening arrhythmias to rule-out potential contributory conditions such as regurgitant or obstructive lesions. Occasionally, the work-up reveals conditions that should be addressed by transcatheter or surgical interventions. For example, in patients with univentricular physiology, the extracardiac conduit is associated with a lower incidence of atrial arrhythmias, which is thought to reflect, in part, reduced exposure of the right atrium to elevated systemic pressures. Fontan conversion from an atriopulmonary connection to a total cavopulmonary connection combined with arrhythmia operation in patients with symptomatic and uncontrollable arrhythmia episodes is associated with significant functional improvement [24].

#### 2.2.3. Myocardial Ischemia

Ischemia is a well-known trigger for cardiac arrhythmias: more than 5% of patients with ST segment elevation myocardial infarction develop ventricular fibrillation [25]. The risk of arrhythmias induced by ischemia varies according to the type of cardiac defect. Coronary artery abnormalities, such as anomalous connection of a coronary artery or coronary artery fistula, are more frequent in patients with CHD. Cases of sudden death have been reported in patients with a left coronary artery that courses between the aorta and the pulmonary artery. Deaths predominantly occur during or just after vigorous exercise, likely due to ischemia resulting from extrinsic coronary artery compression. Other rare coronary abnormalities include the presence of a single coronary artery, coronary atresia, congenital stenosis or atresia of a coronary ostium, and coronary arteries arising from the pulmonary artery. Long-term coronary complications are rare but can also occur in patients with coronary artery reimplantation, such as patients with D-TGA after arterial switch operation following growth and development [26]. In patients with D-TGA and atrial switch surgery, myocardial perfusion defects have also been described [27]. The branching pattern of the major coronary vessels is often abnormal, and hypertrophy of the right ventricle develops over years of exposure to systemic pressures. This inevitably increases myocardial oxygen demand that may exceed supply from a single right coronary artery, particularly during rapid heart rates [28,29].

Paralleling the rising prevalence of traditional cardiovascular risk factors in the aging population of patients with CHD is a higher burden of coronary artery disease [30]. The incidence of myocardial infarction has been reported to be greater than in the general population, and associated with higher mortality [31]. Atherosclerosis may contribute to arrhythmia vulnerability in patients with CHD, and its optimal screening and management should be integrated into a global approach to the CHD patient that considers all potential co-existing conditions.

### 2.3. Modulators for Arrhythmias in Congenital Heart Diseases

#### 2.3.1. Neurohormonal Perturbations

The autonomic nervous system plays an important role in the modulation of arrhythmogenesis. Sympathetic influences on cardiac electrophysiology are complex. Although sympathetic stimulation has similar effects on both atrial and ventricular myocytes, vagal stimulation does not. In the ventricles, vagal stimulation prolongs the action potential duration and effective refractory period, whereas in the atria, vagal activation reduces the atrial effective refractory period, augments spatial electrophysiological heterogeneity, and promotes EADs toward the end of phase 3 of the action potential. This differential effect may explain why parasympathetic stimulation is proarrhythmic in the atria but antiarrhythmic in the ventricles, whereas sympathetic stimulation seems to be proarrhythmic for both chambers [32]. Neurohormonal activation has been reported in different forms of CHD, with increased circulating concentrations of atrial natriuretic peptide, brain natriuretic peptide, endothelin-1 renin, aldosterone, norepinephrine, and epinephrine [33]. Even though asymptomatic subjects had evidence of significant neurohormonal activation, a stepwise increase of these chemical messengers was observed according to functional status and other clinical indices. Interestingly, the level of neurohormonal activation also correlated with electrocardiographic markers, such as QRS duration and QT interval. However, it remains to be demonstrated whether neurohormonal activation has prognostic implications in adults with CHD and whether pharmacological manipulation of neurohormonal systems translates into a clinical benefit on arrhythmia burden.

#### 2.3.2. Chronic Inflammation

Chronic low-level inflammation has increasingly been implicated in cardiovascular disease. Recent evidence demonstrates that medications targeting the inflammatory response can prevent cardiovascular events [34]. The level of high-sensitivity C-reactive protein has been strongly associated with outcomes in adult CHD patients. In a prospective cohort of 707 outpatients, those with the highest quartile of high-sensitivity C-reactive protein more often experienced the combined outcome of all-cause mortality or non-elective cardiovascular hospitalization (30.5% vs. 11.3%, HR = 2.00, 95% CI 1.35–2.97), all-cause mortality (11.9% vs. 1.5%, HR = 4.23, 95% CI 1.87–9.59), and arrhythmic events (HR ~ 2) during an average follow-up of 815 days [35]. These findings implicate inflammation in the pathophysiology of arrhythmia development among adults with CHD. While the underlying mechanisms involved require further study, a better understanding of the causes of inflammation and pathways by which inflammation results in adverse outcomes could potentially identify promising pharmacologic targets.

## 3. Management of Arrhythmias in Congenital Heart Disease

The management of arrhythmias in patients with CHD requires an inter-disciplinary collaboration of cardiologists, surgeons, and electrophysiologists with specific expertise in the care of adults with CHD.

### 3.1. Arrhythmia Diagnosis

In patients with paroxysmal episodes of palpitations or other symptoms suggestive of possible heart rhythm disorders (e.g., faintness or syncope), obtaining a recording of the arrhythmia is the first essential step. The type of exploration depends on the frequency of symptoms, and while repeated ECGs or Holter-ECGs can bring the diagnosis, the emergence of new diagnostic tools are of particular interest in patients with rarer episodes. Implantable loop recorders have, for example, demonstrated a high proportion of arrhythmias in CHD patients with prior negative Holter/event monitoring [36]. Furthermore, the use of connected watches or other digital devices for heart rhythm monitoring (handheld or wearable devices) is rapidly spreading. Most of them provide single-lead recordings, but up to six leads are available in some recent devices. Preliminary data demonstrated the reliability of smart watches in patients with CHD, although the P-waves visualization is sometimes difficult to differentiate the exact type of arrhythmia [37]. In patients without recordable arrhythmia despite the use of these different tools, an electrophysiological study can be considered. This invasive strategy is mainly reserved for CHD patients with a high suspicion of arrhythmia, in particular, when a ventricular arrhythmia is suspected. The prognostic value of programmed ventricular stimulation in risk-stratifying patients has been primarily demonstrated in patients with tetralogy of Fallot [38].

### 3.2. Medical Therapy

The experience with pharmacological therapy in CHD has generally been discouraging, resulting in a growing preference for interventional approaches in most centers and in the latest guidelines [39]. For atrial arrhythmias, a rhythm control strategy (maintenance of sinus rhythm) is generally preferred as a first-line approach, due to the hemodynamic consequences that could result from tachycardia and loss of atrial systole, particular in those with moderate or complex forms of CHD. Sudden death has been reported as a result of rapidly conducting atrial tachyarrhythmias in patients with systemic right ventricles and univentricular hearts [40]. There is a paucity of data to guide therapeutic decisions regarding optimal pharmacological therapy in patients with CHD, with approaches primarily extrapolated from data reported in patients with acquired cardiomyopathies. The selection of antiarrhythmic agents should consider coexisting sinus node or AV node disease, heart failure, associated therapies, child-bearing potential, and comorbidities [41,42]. The use of Class I drugs is discouraged in patients with CHD who have coronary artery disease or systemic and/or subpulmonary dysfunction, as they have been associated with increased mortality in the setting of ventricular scarring. Proarrhythmic effects of antiarrhythmic drugs are poorly studied in patients with CHD. Concerns include residual hemodynamic lesions, incisional scars, intracardiac baffles, conduits, and/or extensive areas of myocardial fibrosis that may predispose to potentially fatal proarrhythmic effects from Class I agents by facilitation of reentrant tachycardias due to decreased conduction velocity and spatially heterogeneous action potential prolongation [43]. Sotalol is an alternative option, but its use has been relegated to a class IIb indication as a first-line agent, given concerns over proarrhythmic effects and meta-analyses reporting increased all-cause mortality in the general population [44]. Amiodarone is the most effective agent and is considered a drug of choice in the setting of heart failure, but long-term administration is limited by time- and dose-dependent side effects. Moreover, the risk of amiodarone-induced thyrotoxicosis is between 4- and 7-fold higher in patients with cyanotic CHD and Fontan palliation [45]. Dofetilide is another potential class III agent that is a reasonable alternative to amiodarone in adults with CHD and ventricular dysfunction [46], but requires a strict protocol of initiation and hospitalization owing to the risk of life-threatening torsade de pointes associated with excessive QT prolongation. It is, moreover, unavailable in many countries worldwide.

### 3.3. Catheter Ablation

Considering the limited efficacy of pharmacological agents and relatively young age of the patient population, catheter ablation has increasingly been used as first-line therapy for a variety of arrhythmias in patients with CHD.

#### 3.3.1. Current Approaches and Outcomes

**Figure 2:**
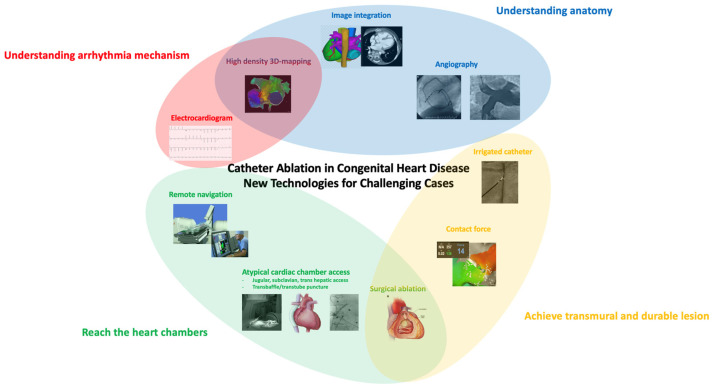
Catheter ablation in congenital heart diseases, technological advances for challenging cases.

The advent of three-dimensional electroanatomic mapping systems and advances in ablative techniques have resulted in significant improvement in outcomes (Figure 2) [47,48]. Target sites for ablation are selected by combining voltage mapping, which localizes areas of scar tissue, activation mapping and pacing maneuvers to elucidate arrhythmia mechanisms and localize critical components of the arrhythmia substrate. The use of irrigated catheter tips has also been associated with more favorable acute outcomes. This may reflect the difficulties in creating transmural lesions in low-flow environments (which impair conductive cooling and limit power delivery of radiofrequency energy), as observed experimentally in certain types of surgical anatomies [49], as well as chronic volume and pressure loads that result in marked thickening of myocardial walls [50]. Image integration combining pre-procedural high-resolution computed tomography or magnetic resonance imaging with electroanatomic maps also facilitates a more thorough understanding of complex anatomical details. These imaging techniques may prove complementary information regarding structural substrates to target.

In patients with intra-atrial reentrant tachycardias (IART), the most common type of arrhythmias in patients with CHD, linear lesions are created to target an identified critical isthmus. The acute success rate exceeds 80% [41,51]. However, recurrent arrhythmias remain common in this population, in particular, in complex substrates such as univentricular hearts and Fontan palliation. More often than not, different circuits or mechanisms are at play suggesting that they more likely result from progressive atrial myopathy as opposed to non-durable prior ablation lesions. Emerging data also suggest the interest of systematically targeting all inducible arrhythmias, whether clinically documented or not, to improve long-term outcomes [52]. Despite recurrent arrhythmias, a significant proportion of patients remain in sinus rhythm. Even if arrhythmias are not entirely abolished by ablation, the procedure often provides substantial improvement in the frequency and duration of episodes and can decrease or eliminate the need for ongoing drug therapy.

In some specific situations, ablation outcomes for tachyarrhythmias remain inferior to those reported in non-CHD patients. For example, in Ebstein anomaly, ablation is complicated by different factors, including atypical accessory pathways with an oblique orientation or multiple insertions, difficulty identifying the true AV groove, dilated cardiac chambers that cause catheter tip instability, and complex electrogram analysis due to low-amplitude fractionated signals recorded from the atrialized right ventricle [53]. The recurrence rate after a first procedure has been reported to be 20% to 40% [54]. In certain cases, right coronary angiography can be performed during the ablation procedure to identify the true location of the tricuspid annulus. Placement of a microelectrode catheter in the right coronary artery has also been reported to map the right AV groove when electrogram patterns are difficult to decipher.

At the ventricular level, when reentrant ventricular tachycardias involve specific anatomically defined narrow conduction corridors, catheter ablation is also associated with favorable acute success rate and long-term freedom from recurrence [55]. In high-risk patients, although catheter ablation of ventricular arrhythmias is not generally considered a substitute for an implantable cardioverter-defibrillator (ICD), catheter ablation without back-up ICD may be discussed in some patients with tetralogy of Fallot to limit the considerable burden of long-term complications associated with ICDs, when performed in highly experienced centers with optimal ablation endpoints reached. Ventricular arrhythmias can also develop independently of direct surgical scarring whenever long-standing hemodynamic overload causes advanced degrees of ventricle dysfunction or hypertrophy. Aortic valve disease, TGA with a systemic right ventricle, severe Ebstein anomaly, and unrepaired tetralogy of Fallot are examples of types of CHD that can eventually lead to this scenario. The global experience with catheter ablation of ventricular arrhythmias in CHD lesions other than tetralogy of Fallot remains scarce.

#### 3.3.2. Technical Challenges to Reach Heart Chambers

**Figure 3:**
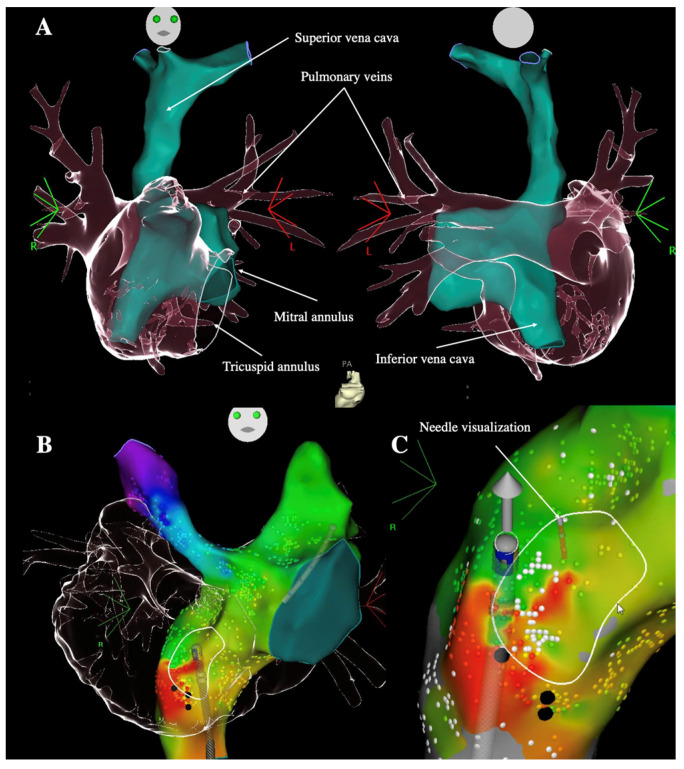
Transbaffle puncture in patients with D-TGA and atrial switch. (**A**) CT-scan images of a patient with Senning surgery in right anterior oblique 30° (left) and posteroanterior view (right) showing that the systemic venous atrium is surrounded by the pulmonary venous atrium. In another patient with Mustard surgery, after the merging of electroanatomical and CT-scan images, the optimal site for transbaffle puncture is located (white circle, (**B**)) and the transseptal needle is connected to be visualized (**C**) (anteroposterior views).

Access issues to the arrhythmia substrate must be carefully considered prior to embarking on a catheter ablation procedure. In cases of occluded veins due to vascular anomalies or prior interventions, jugular, subclavian, or in rare instances transhepatic access can be planned. In complex CHD, accessing the pulmonary venous atrium in distorted anatomies with altered landmarks can be challenging. In patients with an atrial switch operation or total cavopulmonary connection, a retrograde approach using a remote magnetic navigation system is often the preferred approach in centers with access to and high-volume experience with the use of such technology. Remote magnetic navigation increases catheter maneuverability and stability and allows precise delineation of the extracardiac and intracardiac anatomy. Steering the magnetic ablation catheter via the distal end allows any given site within a given cardiac chamber to be reached, thereby increasing mapping and ablation accuracy [56]. In experienced centers where magnetic navigation is not an option, transbaffle or transtube access is used with a high-success rate and low rate of complications. The role of 3D-image integration is essential in those cases, and puncture can be safely performed after incorporation of biplane angiograms or careful fusion of CT-scan or magnetic resonance reconstructions with electroanatomical mapping images to identify the best site for puncture, with or without transesophageal or intracardiac echocardiography guidance [57]. The transseptal needle and guidewire can also be connected to the mapping system for three-dimensional visualization to maximize safety and decrease fluoroscopy use (Figure 3) [58]. The additional use of radiofrequency needles and balloon dilatation may assist in perforating frequently fibrotic and calcified baffles or tubes and to smoothen access with the sheath through the puncture site. Other creative methods, such as transthoracic direct percutaneous access or surgical and interventionnal hybrid procedures, have been described, but are best reserved for carefully selected cases. It remains to be determined whether noninvasive treatment of arrhythmias by the recently described technique of radioablation could be of value in patients with CHD [59].

#### 3.3.3. Perioperative Evaluation of Arrhythmias

Another important issue in CHD patients is the perioperative evaluation of arrhythmia, to (i) guide surgical ablation at the time of concomitant cardiac surgery, or (ii) to treat arrhythmia substrates that become inaccessible after surgery [41,42]. Many centers consider a comprehensive electrophysiology study before referring patients with Ebstein anomaly to surgery. The absence of clear ventricular pre-excitation on the electrocardiogram does not rule out the possibility of a concealed retrograde accessory pathways that could become problematic after surgery. In case of cone reconstruction, the annuloplasty ring often required to reduce the diameter of the tricuspid valve can act as a mechanical barrier to catheter access, and if plication of atrialized right ventricle is performed, areas of ventricular myocardium could also become inaccessible. Moreover, in rare instances when accessory pathways are mapped pre-operatively but cannot be successfully ablated, surgical ablation under direct vision can be attempted [6].

**Figure 4:**
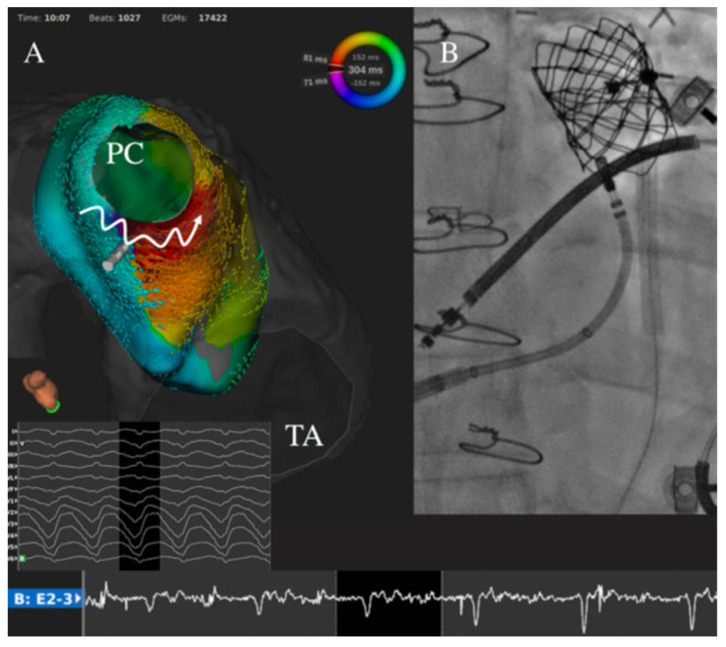
Ventricular tachycardia catheter ablation in a patient with tetralogy of Fallot with transcatheter pulmonary valve. The clinical ventricular tachycardia (cycle length 301 ms, 198 bpm) was easily inducible. Very-high density mapping (Rhythmia system, Boston Scientific, USA) revealed that circuit rotated around the pulmonary valve with the critical isthmus identified between the right ventricle to pulmonary artery conduit and the ventricular septal defect patch (**A**). A linear ablation by irrigated radiofrequency (50 W) slowed and then terminated the arrhythmia. Despite complementary applications including with retrograde aortic approach, a complete bidirectional block was not achieved as the superior part of the isthmus was covered by the transcatheter pulmonary valve (**B**). Reproduced with permission from Combes et al. [60].

**Figure 5:**
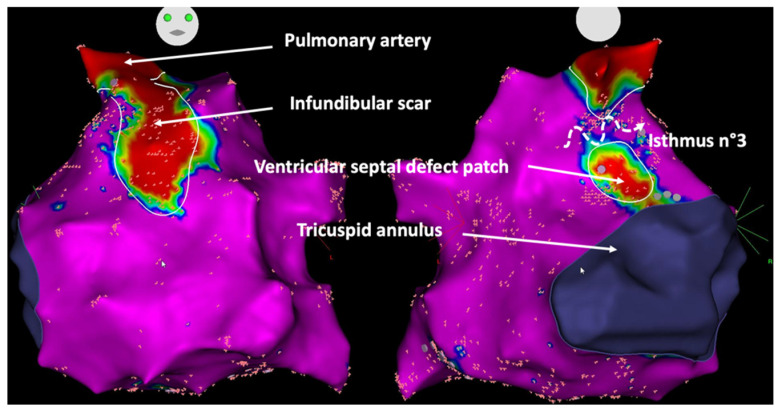
Critical isthmus between ventricular septal defect patch and pulmonary artery in a patient with repaired tetralogy of Fallot. Voltage maps in anteroposterior (**left** Panel) and posteroanterior (**right** Panel) views. Healthy tissue is depicted in purple, scar tissue in red. The isthmus n°3 (curved arrow), between the ventricular septal defect patch and the pulmonary artery, is the critical isthmus most commonly involved in ventricular tachycardias in patients with tetralogy of Fallot. Fragmented potential with slow electrical conduction velocity (<0.5 m/s) are associated with an increased risk of ventricular arrhythmias.

Furthermore, ongoing studies are assessing the value of systematic electrophysiological studies before pulmonary valve replacement in patients with tetralogy of Fallot. At this time, this approach is mainly recommended in patients with a history of sustained ventricular tachycardia [39]. The rationale is that these patients have established risk factors for ventricular arrhythmias, the pulmonary homograft may cover parts of the infundibular septum, preventing subsequent isthmus transection by catheter ablation [60] (Figure 4), and transmural surgical ablation lines can be performed if a critical isthmus is identified during preoperative mapping. Detailed electroanatomic mapping studies revealed specific characteristics of abnormal isthmuses related to ventricular tachycardia in tetralogy of Fallot, such as longer and narrower dimensions, and slower conduction velocities (<0.5 m/s) (Figure 5) [10]. Patients without a slow conducting isthmus at baseline or after ablation appear to have a very low risk of developing ventricular arrhythmias during short-term follow-up. In these patients, RV overload induced by pulmonary regurgitation may act as an important trigger for arrhythmias [61]. In an animal model of physiologic sequelae after tetralogy of Fallot repair, increased RV end-diastolic pressure was for example associated with an increased incidence of inducible atrial and ventricular arrhythmias [62]. Chronic RV overload and stretching also result in progressive myocardial remodeling and fibrosis development associated with a greater risk of ventricular arrhythmias [63]. As recent data suggested that hemodynamic optimization provided by pulmonary valve replacement may be associated with a decrease in ventricular arrhythmias burden in this population [64], substrate-based catheter ablation during systematic electrophysiology study in this clinical situation may further reduce the long-term arrhythmic risk in these patients. The precise evaluation of structural abnormalities with a significant hemodynamic effect that may constitute reversible causes of arrhythmias is of particular importance, and recent advances in imaging, especially magnetic resonance imaging, may help guide decisions regarding optimal timing and indications for reinterventions [61].

#### 3.3.4. Atrial Fibrillation

Understanding electrophysiological mechanisms underlying arrhythmia initiation and sustainability in patients with CHD is an important area of research. In particular, the prevalence of atrial fibrillation is increasing exponentially in this expanding and aging population, and is considered the next arrhythmic epidemic to strike patients with CHD [65]. While most triggers for paroxysmal atrial fibrillation in structurally normal hearts arise from pulmonary veins, caution is warranted in extrapolating mechanistic studies to the CHD population considering the unique anatomical and physiological features. Moreover, as extra-pulmonary triggers are more frequently reported in patients with a high degree of atrial remodeling, the importance of pulmonary vein sources in the genesis of atrial fibrillation remains uncertain in adults with CHD [66]. The largest retrospective series of catheter ablation for atrial fibrillation included 84 patients with various forms of CHD, half of whom had non-paroxysmal atrial fibrillation [67]. Ablation strategies were based mainly on pulmonary vein isolation. At the discretion of the operator, additional linear ablation, non-PV triggers, or complex fractionated atrial electrograms could be targeted. One-year freedom off (53.1%) or on (71.6%) antiarrhythmic drugs was comparable to historical reports of patients without CHD. However, few studies have provided a comprehensive electrophysiological description of atrial fibrillation in patients with CHD. A report of detailed mapping in two individuals provided provocative observations regarding the presence of focal drivers for atrial fibrillation in some patients with CHD [68]. Circumscribed areas exhibiting continuous electrical activity coexisted with parts of the atrium activated in a regular manner. Radiofrequency ablation at these sites terminated atrial fibrillation demonstrating that, at least in some patients, atrial fibrillation recorded on the surface electrogram may result from focal activity giving rise to fibrillatory conduction. The growing literature on atrial fibrillation ablation supports feasibility and safety in patients with CHD, although the data is currently insufficient to claim equivalent outcomes to the non-CHD population. A greater appreciation of underlying mechanisms and substrates to target may contribute substantially to further improving outcomes, in particular, the importance of focal and reentrant non-pulmonary vein triggers is a topic that remains ripe for research and merits formal assessment on a large scale.

## 4. Conclusions

Multiple factors including distorted native and post-surgical anatomies, surgical scarring, and hemodynamic sequelae contribute to structural and electrical remodeling and, together, create unique substrates for arrhythmias in patients with CHD. New ablative technologies have emerged and represent effective therapeutic options for the majority of patients. While most arrhythmias involve macro reentrant circuits, atrial fibrillation is on the rise in this aging population. Much remains to be elucidated regarding underlying mechanisms for atrial fibrillation and substrates to targets. The importance of perioperative electrophysiological evaluation is recognized in some clinical scenarios that may allow for tailored risk stratification and preventive substrate ablation. A continuous and close collaboration between cardiologists, surgeons, and electrophysiologists with expertise in CHD is essential to optimizing outcomes in this challenging patient population.

## Figures and Tables

**Table 1:** Arrhythmias pathophysiology in congenital heart disease.

Substrate and Trigger	Pathophysiology	Comments
CHD-related substrate	abnormal/displaced conduction pathways accessory pathways twin AV nodes	displaced conduction pathways mainly in ccTGA, AVSD, univentricular hearts and heterotaxy syndromes Ebstein anomaly associated with a high prevalence of accessory pathways (often multiple) twin AV nodes mainly reported in AV discordance, AVSD and right or left isomerism
post-operative substrate	incisional flutter around right lateral atriotomy is the second most common mechanism (behind the peritricuspid circuit)∙ incisional flutter around right lateral atriotomy is the second most common mechanism (behind the peritricuspid circuit) wide variety of circuits according to the underlying phenotype and previous surgeries ventricutolomy incisions, patches and conduits can favor the occurrence of ventricular arrhythmias four main anatomical isthmuses described in tetralogy of Fallot	advances in surgical techniques aim to reduce the number or the arrhythmogenesis of incisions
cardiovascular risk-related substrate	aging of the CHD population associated with an increase in the prevalence of the main cardiovascular risk factors	importance of screening and optimal management of associated conditions to decrease the burden of arrhythmia
genetic substrate	important role of genetics in the pathogenesis of CHD genetic abnormalities associated with an increased risk of arrhythmias Several genes identified (e.g., NKX 2.5)	
triggers for arrhythmias	abnormal automaticity and triggered activity hemodynamic alterations myocardial ischemia neurohormonal perturbations chronic inflammation	the importance of remodeling in CHD mays be associated with increased abnormal automaticity and/or afterdepolarizations rule-out hemodynamic conditions (regurgitant or obstructive lesions) in patients with new-onset or worsening arrhythmias coronary artery abnormalities or acquired coronary artery disease neurohormonal activation reported in different forms of CHD C-reactive protein associated with arrhythmic events in CHD

AV, atrioventricular; AVSD, atrioventricular septal defect; ccTGA; congenitally corrected transposition of the great arteries; CHD, congenital heart disease.

**Table 2:** Management of arrhythmias in congenital heart disease.

Item	Management	Comments
diagnosis	ECG Holter-ECG event recorder connected devices (smart watch, handheld ECG) implantable loop recorder electrophysiological study	choice of the diagnostic tool tailored to the patient according to symptom duration and frequency, local infrastructure and patient’s preference
medical therapy	rhythm control strategy preferred modest efficacy data primarily extrapolated from acquired cardiomyopathies class I drugs generally discouraged amiodarone associated with a high burden of long-term side effects	antiarrhythmic agents should consider coexisting sinus node or AV node disease, heart failure, associated therapies, child-bearing potential, and comorbidities
catheter ablation	advances in technologies and growing experience importance of high-density mapping and image integration significant improvement of outcomes should be considered as first-line therapy long-term recurrences remain common interest of targeting all inducible arrhythmias global experience with catheter ablation of ventricular arrhythmia in CHD other than tetralogy of Fallot remains scarce underlying mechanisms and substrates to target in atrial fibrillation are key research priorities	catheter ablation must be performed in expert centers with multidisciplinary specialized teams remote magnetic navigation of particular interest in complex anatomies transbaffle or transtube punctures used with high-success rate and low rate of complications when performed by experienced operators
perioperative assessment	to treat arrhythmia substrate that will become inaccessible after surgery or to guide surgical ablation in Ebstein patients before surgery (at least in patients with ventricular pre-excitation and systematically in some teams) in Fallot patients before pulmonary valve replacement, electrophysiology study +/− catheter ablation now recommended in patients with history of sustained VT	ongoing studies are assessing the value of systematic electrophysiological studies before pulmonary valve replacement in tetralogy of Fallot (in the absence of documented arrhythmia) and the interest of prophylactic catheter ablation of potential critical isthmuses

AV, atrioventricular; CHD, congenital heart disease; ECG, electrocardiogram; VT, ventricular tachycardia.

## Data Availability

Not applicable.
